# Trends in the epidemiology of cardiovascular disease in the UK

**DOI:** 10.1136/heartjnl-2016-309573

**Published:** 2016-08-22

**Authors:** Prachi Bhatnagar, Kremlin Wickramasinghe, Elizabeth Wilkins, Nick Townsend

**Affiliations:** Nuffield Department of Population Health, British Heart Foundation Centre on Population Approaches for Non-Communicable Disease Prevention, University of Oxford, Oxford, UK

**Keywords:** Stroke

## Abstract

Cardiovascular disease (CVD) mortality in the UK is declining; however, CVD burden comes not only from deaths, but also from those living with the disease. This review uses national datasets with multiple years of data to present secular trends in mortality, morbidity, and treatment for all CVD and specific subtypes within the UK. We produced all-ages and premature age-standardised mortality rates by gender, standardised to the 2013 European Standard Population, using data from the national statistics agencies of the UK. We obtained data on hospital admissions from the National Health Service records, using the main diagnosis. Prevalence data come from the Quality and Outcome Framework and national surveys. Total CVD mortality declined by 68% between 1980 and 2013 in the UK. Similar decreases were seen for coronary heart disease and stroke. Coronary heart disease prevalence has remained constant at around 3% in England and 4% in Scotland, Wales, and Northern Ireland. Hospital admissions for all CVD increased by over 46 000 between 2010/2011 and 2013/2014, with more than 36 500 of these increased admissions for men. Hospital admission trends vary by country and CVD condition. CVD prescriptions and operations have increased over the last decade. CVD mortality has declined notably for both men and women while hospital admissions have increased. CVD prevalence shows little evidence of change. This review highlights that improvements in the burden of CVD have not occurred equally between the four constituent countries of the UK, or between men and women.

## Introduction

In 2014, cardiovascular disease (CVD) was the second main cause of death in the UK.^1^ The Global Burden of Disease (GBD) study has shown that the burden of CVD is declining in the UK;^2^ between 1990 and 2013 CVD death rates in England declined by 52%, coronary heart disease (CHD) by 60%, and stroke by 46%. The GBD study compares mortality, disability adjusted life-years, years of life lost, and years lived with disability between 1990 and 2013 for England and its regions, but the GBD visualisation tool also provides this information for Wales, Scotland, and Northern Ireland. To further understand the burden of CVD in the UK, we aim to report trends in age-standardised mortality, prevalence, and treatment rates for CVD, CHD, and stroke for England, Wales, Scotland, and Northern Ireland over a number of years.

Previous studies have reported changes in CVD incidence, prevalence, and treatment. A study linking hospital admissions and mortality data showed that within England, a 50% decline in age-standardised mortality from myocardial infarction was due to both a reduction in incidence and case fatality.^3^ These changes in mortality and incidence are likely to have affected treatment and the prevalence of CVD, especially its two most common subtypes, CHD and stroke. A study using the General Practice Research Database (GPRD) compared prevalence of CHD in 1999 and 2007 in England in those aged over 25 years^4^ and found a decrease in the prevalence of CHD, from 1.74 million CHD patients in 1999 to 1.53 million CHD patients in 2007. The British Regional Heart Study (BRHS) reports that in men aged 40–59 years there was no clear trend between 1979 and 1996 in the diagnosis of CHD.^5^

This review is based on the 2015 *Cardiovascular Disease Statistics* report published for the British Heart Foundation; although there are more detailed and online supplementary data presented in this review, we use national datasets that are collected annually to allow patterns in trends over time to be observed, rather than comparing two separate years as has been done previously. When possible, we have presented this trend data by gender, for the UK as a whole and for England, Scotland, Wales, and Northern Ireland separately, resulting in a more detailed observation of secular trends in the burden of CVD in the UK than has been previously presented. We present mortality trends that are age-standardised to the 2013 European Standard Population (ESP) for the first time, an update from the 1976 ESP. We also present trends in different measures of prevalence, hospital admissions, and treatment for CVD, CHD, and stroke.

10.1136/heartjnl-2016-309573.supp1 Supplementary tables

## Methods

We present trends in mortality from the introduction of ICD 9, the ninth International Classification of Diseases (1979), through to the latest year available at the time of writing. Trends in hospital admissions, prevalence, and treatment data are reported from the earliest year available.

We obtained mortality and population data by 5 year age group and sex on total CVD, CHD, and stroke from the national statistics agencies of the UK. Using this, we produced all ages and under 75 age-standardised mortality rates by gender, directly age-standardised to the 2013 ESP. Stata V.13 was used to age-standardise the mortality data.

We present prevalence data from three sources on which no extra analyses were performed. The Quality and Outcomes Framework (QOF) is collected annually through general practice records. The QOF rewards general practices for keeping records on the number of patients on their registers who have been diagnosed with particular conditions. The Health Surveys of England and Scotland ask specific questions about the prevalence of CVD, CHD, and stroke, although these questions are not asked annually. The General Lifestyle Survey (GLS) ended in 2011 and was an annual cross-sectional survey for Great Britain that included questions on whether people had certain conditions. All three of these surveys are sampled so they are nationally representative and all collect self-reported measures on the prevalence of CVD through household interviews.

We obtained data on hospital admissions from National Health Service records using the main diagnosis attributed to a patient on being discharged from hospital; in the main paper we report finished consultant episodes (FCEs), which include both ordinary admissions and day cases. We refer to FCEs as ‘admissions’ in this review, but it is important to note that FCEs are distinct from ordinary admissions. The online supplementary files contain more detailed FCEs for England only and do not include day cases. Community pharmacist prescriptions data are from Prescriptions Cost Analyses, published by the UK national statistics agencies and health services. Operations data come from the British Cardiovascular Intervention Society and the Society for Cardiothoracic Surgery in Great Britain & Ireland.

## Trends in mortality

**Figure 1:**
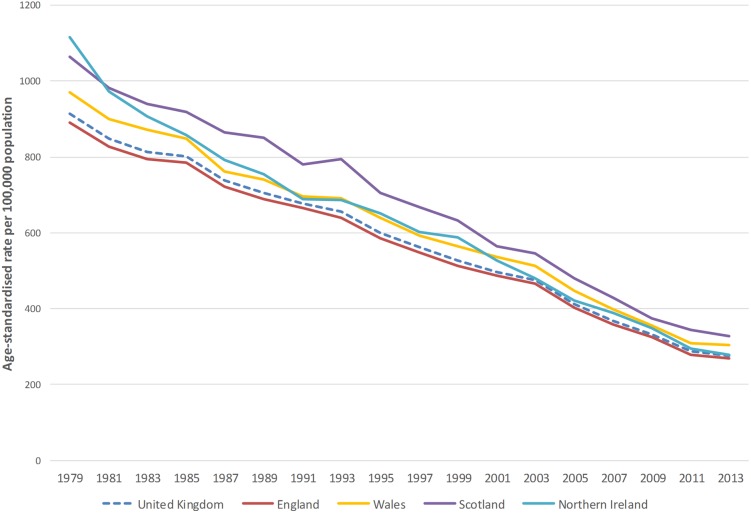
Age-standardised death rates per 100 000 from cardiovascular disease, all ages, UK and England, Wales, Scotland, Northern Ireland, 1979–2013.

**Figure 2:**
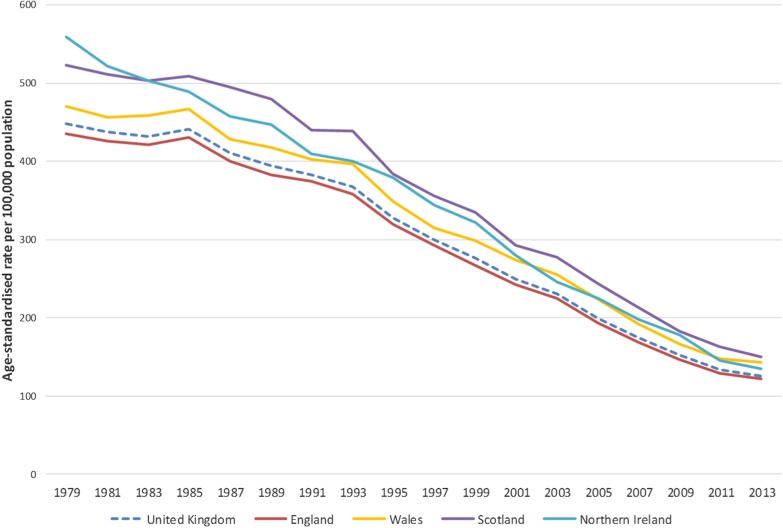
Age-standardised death rates per 100 000 from coronary heart disease, all ages, UK and England, Wales, Scotland, Northern Ireland, 1979–2013.

**Figure 3:**
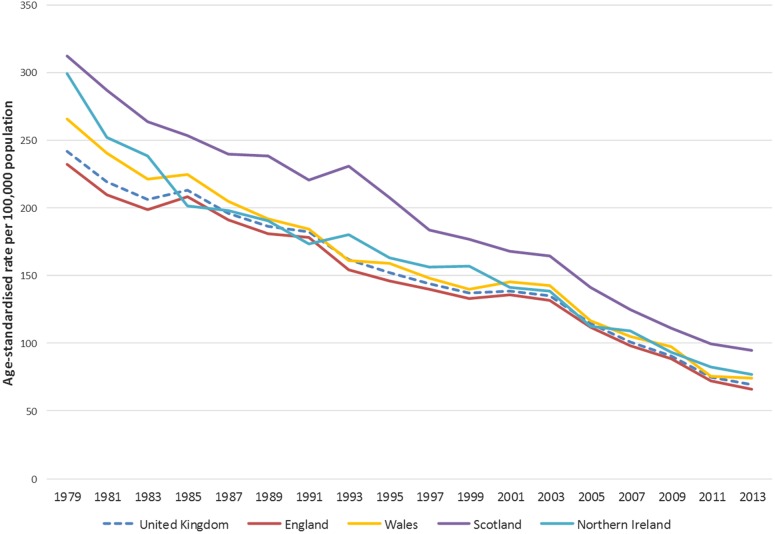
Age-standardised death rates per 100 000 from stroke, all ages, UK and England, Wales, Scotland, Northern Ireland, 1979–2013.

Figures 1–3 show that age-standardised CVD, CHD, and stroke death rates declined notably between 1979 and 2013 in the UK, and that there were persistent differences in the absolute rates between the four countries of the UK. For the UK, total CVD mortality declined by 70% between 1979 and 2013, CHD mortality decreased by 72%, and mortality from stroke decreased by 71% between 1979 and 2013 (data available in online supplementary files). Trends in age-standardised premature mortality (aged under 75 years) from CVD, CHD, and stroke mortality are presented in the online supplementary tables and show that there were large decreases in premature mortality from these conditions between 1979 and 2013. There was a 78% reduction in premature CVD mortality, 81% decrease in premature CHD mortality, and an 80% decline in premature stroke mortality.

Between 1979 and 2013, total CVD mortality decreased by 70% in England and 69% in Wales and Scotland. Northern Ireland had the largest decline with a 75% decrease in CVD mortality between these years; in 1979 Northern Ireland had the highest CVD death rate in the UK, but by 2013 only England had a lower CVD death rate. CVD mortality in those under 75 years of age follows a similar pattern, with the largest reduction occurring in Northern Ireland at 83%.

CHD mortality at all ages decreased by 72% in England, 70% in Wales, 71% in Scotland, and 76% in Northern Ireland between 1979 and 2013. Premature CHD mortality followed a similar pattern, with the largest declines in Northern Ireland (84%) and the smallest in Wales (79%). The largest decrease was in women in Northern Ireland, with a reduction of 87% for premature CHD mortality between 1979 and 2013.

As with CVD and CHD, there were large declines in total and premature mortality from stroke between 1979 and 2013 for all UK nations. England saw a 71% decrease, Wales 72%, Scotland 70%, and Northern Ireland 74%. The largest decline was for men in Northern Ireland who had a decrease in stroke mortality of 77%. This pattern is mirrored for stroke mortality under 75 with the largest declines in Northern Ireland (84%) and the smallest in Scotland (79%).

## Trends in prevalence

**Figure 4:**
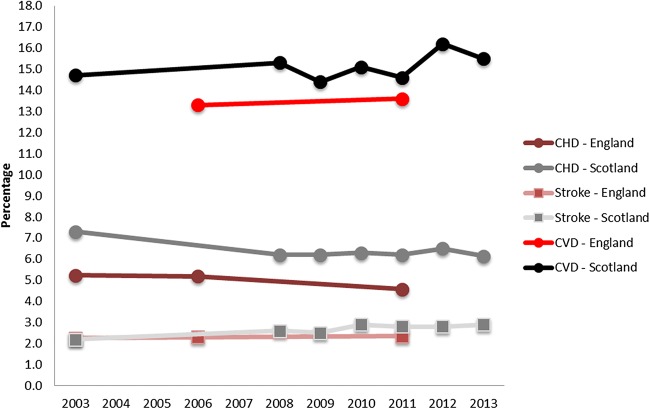
Trends in the prevalence of cardiovascular disease (CVD), coronary heart disease (CHD), and stroke, from the health surveys of England and Scotland 2003–2013.

QOF data show that the prevalence of CHD remained constant at around 3% in England and 4% in Scotland, Wales, and Northern Ireland between 2004/2005 and 2014/2015. The prevalence of stroke has also changed little, remaining at around 2%, although there is an indication of a small increase for England, Scotland, and Northern Ireland (table 1). Figure 4 presents prevalence data from the health surveys for England and Scotland from 2003 to 2013; although the pattern of Scotland having a higher prevalence than England remains, overall the surveys show a higher prevalence than that reported by QOF data.

**Table 1:** Trends in the prevalence of selected cardiovascular conditions from QOF data, England, Wales, Scotland, and Northern Ireland 2004/2005 to 2014/2015

	2004/2005	2005/2006	2006/2007	2007/2008	2008/2009	2009/2010	2010/2011	2011/2012	2012/2013	2013/2014	2014/2015
*Coronary heart disease* (%)											
England	3.6	3.6	3.5	3.5	3.5	3.4	3.4	3.4	3.3	3.3	
Scotland					4.4	4.4	4.4	4.4	4.3	4.3	
Wales			4.3	4.2	4.2	4.1	4.0	4.0	3.9	3.9	
Northern Ireland	4.2	4.2	4.2	4.1	4.1	4.0	4.0	3.9	3.9	3.9	3.8
*Stroke and TIA* (%)											
England	1.5	1.6	1.6	1.6	1.7	1.7	1.7	1.7	1.7	1.7	
Scotland					2.0	2.1	2.1	2.1	2.1	2.2	
Wales			2.0	2.0	2.0	2.0	2.1	2.1	2.0	2.0	
Northern Ireland	1.4	1.6	1.6	1.6	1.7	1.7	1.8	1.8	1.8	1.8	1.8

These data are raw prevalence rates and are not adjusted to account for patient age distribution or other factors that may differ between general practices.

Source: England—Health and Social Care Information Centre. QOF achievement data; Scotland—ISD (Information Services Division) Scotland. QOF achievement data; Wales—StatsWales. QOF achievement data; Health, Social Services and Public Safety (2014). QOF achievement data; Northern Ireland 2014/15. http://www.dhsspsni.gov.uk/index/statistics/qof/qof-achievement.htm (accessed June 2015).

QOF, Quality and Outcomes Framework; TIA, transient ischaemic attack.

As with QOF data, CHD prevalence as measured by health surveys has remained stable with a slight decline since 2003 for both England and Scotland. In the health surveys, stroke prevalence has not increased in England. In Scotland, however, there has been a larger increase than as measured by QOF data. Online supplementary data show trends by gender within England and Scotland. These show that men in England have had a slightly higher increase in stroke prevalence as compared to women. The Scottish Health Survey indicates that CVD prevalence dropped for women in 2008, but not for men. However, CVD prevalence climbed again for women so that 2013 prevalence rates were similar between the two genders.

**Figure 5:**
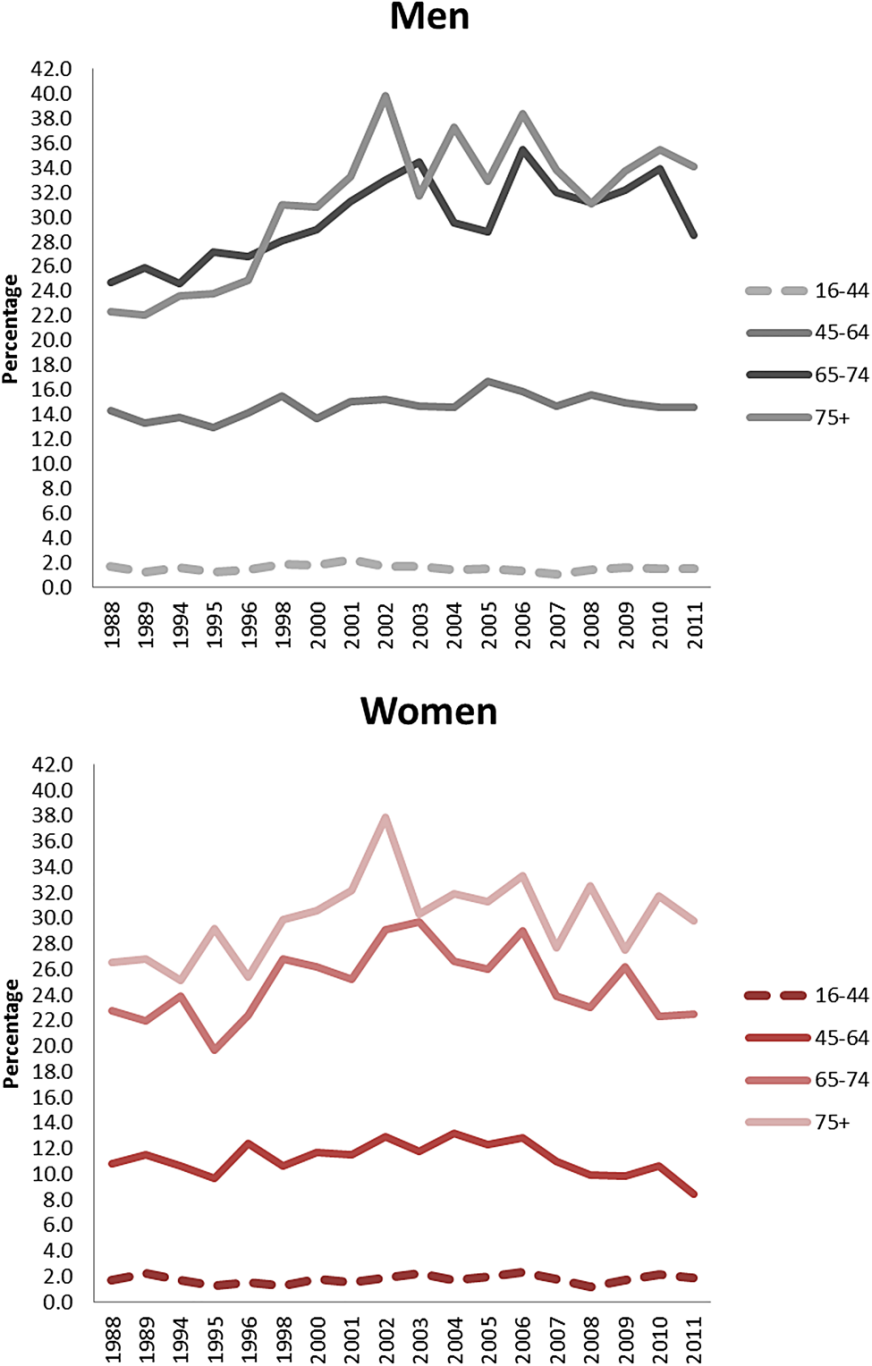
Trends in the prevalence from cardiovascular diseases in men and women by age, from the General Lifestyle Survey, Great Britain 1988–2011.

The GLS has data on CVD prevalence from 1988 to 2011 for Great Britain (figure 5). Although the prevalence is higher than that reported in the health surveys, the GLS provides trends by age and sex. These data suggest that there has been an increase in CVD prevalence for both men and women aged over 75 and for men aged 65–74. CVD prevalence for men aged 16–64 has remained constant, but for women aged 45–64 there was a small decrease from 10.8% to 8.4%.

## Trends in treatment

Table 2 shows that numbers of admissions (finished consultant episodes which include ordinary admissions and day cases) for all CVD increased by over 46 000 between 2010/2011 and 2013/2014 in the UK; however, over 36 500 of these increased admissions were in men (presented in online supplementary tables). Between these years, male admissions for CHD increased by almost 3000, but decreased by around 5000 in women. Stroke admissions remained steady for men and decreased by around 4500 in women.

**Table 2:** Inpatient episodes by main diagnosis in National Health Service hospitals, England, Scotland, Wales, Northern Ireland and UK, 2005/2006 to 2013/2014

	2005/06	2006/07	2007/08	2008/09	2009/10	2010/11	2011/12	2012/13	2013/14
*England*									
All diseases of the circulatory system (CVD) (I00-I99)	1 244 004	1 255 590	1 274 674	1 322 295	1 358 247	1 371 809	1 381 635	1 374 094	1 401 232
Coronary heart disease (I20-I25)	428 262	427 913	424 247	422 334	407 675	405 096	409 508	404 089	401 007
Stroke (I60-I69)	178 321	176 452	179 999	190 101	203 705	198 335	194 436	196 081	197 356
Other CVD	637 421	651 225	670 428	709 860	746 867	768 378	777 691	773 924	802 869
*Scotland*									
All diseases of the circulatory system (CVD) (I00-I99)					144 900	149 032	148 739	148 766	157 298
Coronary heart disease (I20-I25)					47 923	50 157	48 699	47 640	49 615
Stroke (I60-I69)					22 441	22 639	22 662	23 737	19 257
Other CVD					74 536	76 236	77 378	77 389	88 426
*Wales*									
All diseases of the circulatory system (CVD) (I00-I99)	74 824	75 338	75 981	78 714	81 599	80 903	77 589	80 953	88 105
Coronary heart disease (I20-I25)	24 516	25 060	25 156	25 074	24 893	24 300	23 904	24 335	25 647
Stroke (I60-I69)	11 660	11 143	11 188	12 313	12 411	12 471	11 460	12 080	13 265
Other CVD	38 648	39 135	39 637	41 327	44 295	44 132	42 225	44 538	49 193
*Northern Ireland*									
All diseases of the circulatory system (CVD) (I00-I99)						42 887	46 617	42 875	44 296
Coronary heart disease (I20-I25)						14 409	14 060	14 520	15 378
Stroke (I60-I69)						4872	3199	2943	3383
Other CVD						23 606	29 358	25 412	25 535
*UK*									
All diseases of the circulatory system (CVD) (I00-I99)						1 644 631	1 654 580	1 646 688	1 690 931
Coronary heart disease (I20-I25)						493 962	496 171	490 584	491 647
Stroke (I60-I69)						238 317	231 757	234 841	233 261
Other CVD						912 352	926 652	921 263	966 023

Notes: Finished consultant episodes; ordinary admissions and day cases combined. Pregnancy cases not included. ICD-10 codes in parentheses.

Source: Department of Health (2014). Hospital Episode Statistics 2013/2014. http://www.hesonline.nhs.uk (accessed April 2015); Information Services Division Scotland (2014) Main diagnosis discharges from hospital 2013/2014. http://www.isdscotland.org (accessed April 2015). Personal correspondence; NHS Wales Informatics Service (2014). The Patient Episode Database for Wales–2013/2014. http://www.infoandstats.wales.nhs.uk (accessed April 2015; Hospital Information Branch (2014). Northern Ireland Episode Based Acute Inpatient and Day Case Activity Data (2013/2014) http://www.dhsspsni.gov.uk (accessed April 2015). Personal correspondence.

CVD, cardiovascular disease; ICD-10, International Classification of Diseases, 10th revision.

Between 2005/2006 and 2013/2014, both England and Wales experienced an increase in the total number of CVD admissions by 11% and 15%, respectively, but patterns differ for CHD and stroke. Wales experienced a 4% increase in admissions for CHD over this period; however, this was driven entirely by an increase in male admissions, with a 6% decrease in CHD admissions for women. In England, admissions for CHD decreased by 6% overall, with a 4% decrease for men but an 11% decrease for women. Stroke admissions increased in England by 13% in men and by 6% in women. In Wales increases in stroke admissions also differed by gender, with a 7% increase for women but a 17% increase for men.

Data for Scotland are available from 2009/2010 and show a 3% increase in CHD admissions and a 14% decrease in admissions for stroke during this period. Northern Ireland hospital admission data are available between 2010/2011 and 2013/2014 and show a 6% increase in CHD admissions; this was largely driven by an increase in male admissions, with an 8% increase during this period for men, but a 2% increase for women. Stroke admissions in Northern Ireland decreased by 31%, which equates to around 1500 fewer admissions during this period, split fairly evenly between men and women.

Trends in prescriptions for CVD are presented in the online supplementary tables. Between 1991 and 2014 in England the number of prescriptions for diseases of the circulatory system increased by around 243 million or 78%. Between 2005 and 2014, CVD prescriptions in Wales increased by around 5.5 million (23%), by 2.3 million (9%) in Scotland, and by 2.5 million (28%) in Northern Ireland.

**Figure 6:**
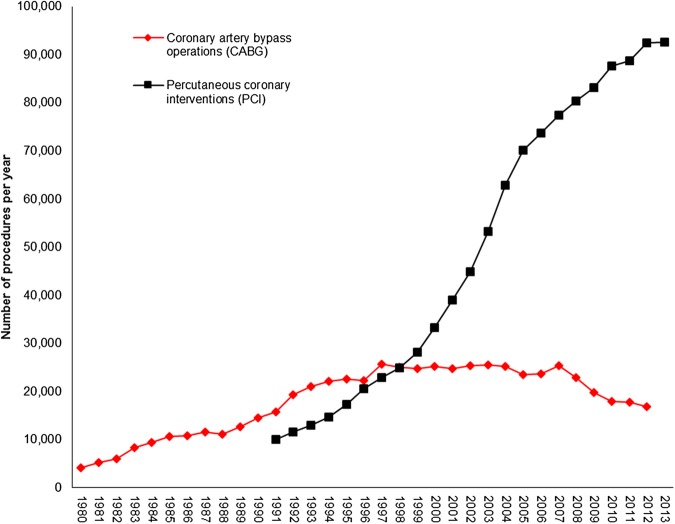
Number of coronary artery bypass operations and percutaneous coronary interventions per year, UK 1980–2013.

The number of surgical interventions for CVD increased between 1980 and 2013 (figure 6). In 2013 there were more than seven times the number of percutaneous coronary interventions (PCI) as compared to two decades earlier (1993). The number of coronary artery bypass grafts reached a peak in the late 1990s but have since decreased by 33%, having been overtaken by PCI as the major coronary surgery.

## Discussion

Age-standardised mortality from CVD, CHD, and stroke has declined by around 70% over the last 30 years, with even larger declines in premature mortality. Northern Ireland has seen the largest decreases of all UK countries. CVD prevalence appears to have increased slightly in England and Scotland, and data for Great Britain shows the largest increases were in men and women aged over 65. CHD prevalence shows evidence of a small decline over the past decade, while stroke prevalence increased slightly in all UK countries. Hospital admissions for CVD increased over the last decade, although patterns differed for CHD and stroke, with increases seen for some UK countries. The numbers of prescriptions and operations for CVD have increased over the last 20–30 years.

The GBD study compared mortality rates in 1990 to those in 2013, and standardised to the GBD 2013 standard population.^2^ When standardised to the 2013 ESP, between the same years our data showed greater decreases in CVD, CHD, and stroke mortality than the GBD; however, the use of a different standard population and slightly different methods may explain the differences. These large declines in CVD, CHD, and stroke mortality are consistent with other countries in both Europe^6^ and the USA.^7^

The BRHS and a GPRD study have both compared CHD prevalence between 2 years to give an estimate of time trends (1979 to 1996 and 1999 to 2007, respectively). The findings from the BRHS are in line with the trends reported here which indicated little evidence of a change in CHD prevalence over the last decade.^5^ The GPRD study reported a decrease in absolute numbers of patients living in the community with CHD.^4^ These two studies use different data sources, age ranges, and time periods, therefore the discrepancy in their findings may be due to these differences. The GPRD study used a sample of general practice records for those aged over 25, whereas the BRHS asked men from 24 British towns in a longitudinal cohort who were aged 40–59 at recruitment to recall if they have ever had a diagnosis of CHD. The age-specific trends presented in figure 2 indicate an increase in CVD prevalence in only the older age groups over the past 20 years, but it is feasible that this trend is driven more by stroke prevalence than CHD prevalence.

A study using the GPRD to assess changes in stroke incidence and prevalence reported a 12.5% increase in stroke prevalence between 1999 and 2008.^8^ These findings are in line with the trends we report in this review, which found an increase in stroke prevalence between 2003 and 2014 using both QOF data and data from the Health Survey for England and the Scottish Health Survey.

We do not report directly on incidence trends in this review, although FCEs can be used as a proxy for incidence. For England, Smolina *et al*^3^ report a 33% and 31% decline in the incidence of myocardial infarction in men and women, respectively, between 2002 and 2010, using hospital episodes linked to mortality data. Although we report on CHD rather than myocardial infarction, trends in FCEs alone also showed a decrease between 2005/2006 and 2013/2014 for men and women in England. Pearson-Stuttard *et al*^4^ compared trends in hospital admissions for acute myocardial infarction between 1999 and 2007 in England and reported a decrease of around 8000 admissions for both men and women. The decreases we report for CHD demonstrate the same pattern in England, with a decrease in FCEs for CHD over the last decade.

Trends in the incidence of stroke have been reported on using the GPRD and an Oxfordshire based primary care dataset. GPRD data compared 1998 and 2008 and showed a 30% decrease in stroke incidence for the UK during this period.^8^ The Oxford Vascular Study reported on stroke incidence in Oxfordshire by comparing rates between 1981 and 1984 with those between 2001 and 2004.^9^ During this 20 year period they report a 29% reduction in stroke incidence (first stroke only), which corroborates the GPRD study findings. The data we present here are for a later time period but also indicate that there was an overall decline in UK stroke admissions.

There were differential trends in stroke admissions between the countries of the UK. England and Wales admissions data are for a longer time period (2005/2006 to 2013/2014), whereas Scotland and Northern Ireland data are from 2009/2010 and 2010/2011, respectively. Using the period between 2010/2011 and 2013/2014 only, stroke admissions in England do show a decline, particularly for women, but a small increase in admissions is still evident for Wales. A 2010 systematic review of stroke incidence in the UK reported that there is limited information on how stroke incidence varies by region of the UK, but that from the five studies included in the review, patterns of incidence rates largely mirror patterns of stroke mortality rates around the UK.^10^ Data in our review demonstrate that stroke mortality rates have decreased. Although 2011 to 2013 only includes 3 years of data, the decrease in stroke mortality between these years was 2% in Wales, compared to 8% in England, 5% in Scotland, and 7% in Northern Ireland, suggesting that a different trend of stroke incidence in Wales may indeed be present for the most recent years.

Despite large decreases in mortality, increases in CVD, CHD, and stroke prevalence were small, with the exception of men over 75 who saw a 10 percentage point increase. Mortality from myocardial infarction has reduced over the last decade; 50% of this decrease is attributed to decreases in incidence and 50% is due to improved case fatality.^3^ Therefore it is possible that the decreased incidence has offset the increases in survival, which would lead to increased prevalence.

There were persistent differences between countries in the overall burden of CVD, with Scotland having a consistently higher burden. Scotland has higher levels of deprivation than the other UK countries, which are a potential explanation for the higher mortality and prevalence levels. The England GBD study showed that different measures of disease burden mirrored deprivation rates.^2^ A pooled analysis of 18 cohort studies found that for excess mortality in Scotland, as compared to England, only 25% was explicable by socioeconomic, behavioural, anthropological or biological risk factors;^11^ therefore higher mortality rates in Scotland may also be due to differences in hospital access, ambulance services or case-fatality rates.

The National Service Framework for CHD outlined priorities to be achieved between 2000 and 2010, which included identifying and treating people at high risk, increasing the use of effective medicines for people following a cardiac event, and increasing the number of revascularisations. The increases seen in prescriptions and revascularisation operations in this review indicate that the implementation of this framework was successful. However, it is important to note that these data do not account for increases in population, which are also likely to be a significant contributing factor. This review on trends in the burden of CVD, CHD, and stroke aimed to include the best available data; there are, however, a number of weaknesses. QOF data may be subject to changes in clinical case finding by general practitioners or differences in clinical coding practices.^12^ Other sources of prevalence data such as the Clinical Practice Research Database may have similar issues, although this dataset only samples 7% of the UK population.^13^ Prevalence reports from surveys face problems of accurately recalling both the type and date of diagnoses. Ideally we would have reported sex-specific trends in prevalence for all data but QOF data are not available by sex. Finished consultant episodes (both ordinary admissions and day cases) are a proxy for incidence as they do not count people who died before reaching hospital and do not distinguish between people with a first event or a recurring event. We were also unable to provide age-standardised hospital admission rates as these data were not available by age group. Consequently, both hospital admissions and all treatment trends do not take account of the increase in population over time. Prescriptions data include prescriptions that may have been provided as secondary prevention for people identified as being at high risk for a CVD event; therefore this should be noted when using the data as a measure of existing burden.

The strengths of this review are that we present the first long-term age-standardised trends in CVD, CHD, and stroke using the 2013 ESP. Despite its limitations, the trend data we present use routinely collected national data and include a number of years, rather than just comparing 2 years, therefore allowing for measurement of changes in trends over time.

## Conclusion

Despite large reductions in mortality from CVD, CHD, and stroke, these conditions have remained a substantial burden to the UK, with rises in treatment and hospital admissions for all CVD. There is some evidence that improvements have not occurred equally for men and women or between the countries of the UK. Although these are promising trends for mortality and stroke admissions in women, prevalence and treatment are increasing over time for all CVD and stroke. Better monitoring of trends in the prevalence and incidence of CVD, CHD, and stroke over time is needed. This review highlights that improvements in the burden of CVD have not occurred equally. Understanding why trends have differed among subpopulations will be valuable for continuing the reduction in the incidence of and mortality from CVD, CHD, and stroke.

**Key messages**

**What is already known about this subject?**

- Cardiovascular disease mortality declined during the 20th century, changing from being the main cause of death to being the second main cause of death in the UK. Coronary heart disease by itself is still the single biggest cause of death in the UK. Overall, cardiovascular disease is still a large burden in the UK.

**What does this study add?**

- We present updated trends in cardiovascular mortality for the UK, standardised to the 2013 European Standard Population. We compare the trends in mortality to trends in prevalence, hospital admissions, and treatments to give an overall view of how the burden of cardiovascular disease has changed over time in the UK.

**How might this impact on clinical practice?**

- It is important for clinicians to understand how the overall burden of cardiovascular disease in the UK is changing over time between men and women, and between England, Scotland, Wales, and Northern Ireland.
