# Supplementary material for: Trends in the epidemiology of cardiovascular disease in the UK
Source: Heart. 2016 Aug 22;102(24):1945–52. doi: 10.1136/heartjnl-2016-309573 (PMC5256396; doi:10.1136/heartjnl-2016-309573)
Supplement: Supplementary tables [file heartjnl-2016-309573supp_tables.pdf]

Age-standardised death rates per 100,000 from cardiovascular disease (CVD), all ages, United Kingdom and England, Wales, Scotland, Northern Ireland, 1979 to 2013

	United Kingdom			England			Wales			Scotland			Northern Ireland		
**Year**	**Men**	**Women**	**Both**	**Men**	**Women**	**Both**	**Men**	**Women**	**Both**	**Men**	**Women**	**Both**	**Men**	**Women**	**Both**
**1979**	1152	753	914	1121	733	890	1229	797	971	1344	889	1063	1435	904	1115
**1981**	1072	695	848	1046	678	828	1137	737	899	1248	814	982	1247	786	974
**1983**	1032	664	814	1008	648	795	1127	701	872	1186	778	939	1142	743	907
**1985**	1014	656	803	994	641	786	1083	688	848	1163	763	920	1062	713	859
**1987**	933	600	737	914	585	721	973	618	762	1097	717	866	991	652	792
**1989**	890	578	706	867	562	688	929	608	740	1076	706	851	963	615	755
**1991**	853	554	677	840	543	665	878	563	695	972	653	781	866	565	690
**1993**	824	537	656	805	521	639	855	568	691	987	663	795	865	558	686
**1995**	753	491	601	737	477	586	799	525	639	868	593	706	811	540	652
**1997**	701	461	562	685	449	548	741	486	592	822	559	668	761	492	602
**1999**	654	434	527	637	421	513	697	464	565	783	527	632	723	493	588
**2001**	613	411	497	603	402	487	657	448	537	684	476	564	648	439	526
**2003**	578	398	475	565	390	466	635	425	513	670	457	546	581	404	479
**2005**	500	345	413	490	337	404	536	376	447	577	406	480	519	350	421
**2007**	443	307	367	432	299	359	473	335	397	527	355	428	477	324	389
**2009**	399	277	331	392	271	325	424	300	356	450	316	375	421	295	350
**2011**	350	236	286	342	229	279	377	253	308	412	288	343	365	239	294
**2013**	333	227	275	325	221	268	377	246	304	392	275	327	336	232	277

Notes: Directly age-standardised to the European Standard Population 2013.

Source: 1969 to 2009: England and Wales, Office for National Statistics (2010) Personal communication.
Scotland; General Register Office (2010) Personal communication.
Northern Ireland, Statistics and Research Agency (2010) Personal communication.
2010 to 2013: England and Wales, Office for National Statistics; Scotland, General Register Office for Scotland; Northern Ireland, Northern Ireland Statistics and Research Agency.

Age-standardised death rates per 100,000 from coronary heart disease (CHD), all ages, United Kingdom and England, Wales, Scotland, Northern Ireland, 1979 to 2013

	United Kingdom			England			Wales			Scotland			Northern Ireland		
**Year**	**Men**	**Women**	**Both**	**Men**	**Women**	**Both**	**Men**	**Women**	**Both**	**Men**	**Women**	**Both**	**Men**	**Women**	**Both**
**1979**	647	314	448	629	305	436	693	323	470	759	374	522	800	394	559
**1981**	626	310	437	611	301	426	664	319	456	722	374	511	747	367	522
**1983**	620	305	432	604	297	421	675	315	458	720	364	503	722	355	504
**1985**	624	316	441	611	308	431	676	328	467	713	378	509	675	360	490
**1987**	581	295	411	567	286	400	615	304	429	696	366	494	637	334	458
**1989**	552	287	395	537	278	383	587	303	418	665	360	479	628	327	448
**1991**	532	280	382	522	273	374	563	291	402	608	331	440	574	297	409
**1993**	510	269	368	498	260	358	548	291	396	599	331	439	552	294	400
**1995**	458	238	328	447	231	320	489	252	349	525	288	383	515	288	380
**1997**	417	218	300	407	212	292	447	225	315	483	269	355	477	251	344
**1999**	383	200	276	372	193	267	418	212	299	460	250	335	442	238	322
**2001**	345	180	249	338	174	242	376	200	274	394	222	293	380	209	279
**2003**	317	169	231	308	163	225	352	186	255	383	205	278	335	184	246
**2005**	275	144	199	266	138	193	307	162	224	332	180	244	312	164	225
**2007**	240	124	174	232	120	168	260	140	192	298	152	213	274	143	198
**2009**	211	107	152	205	102	147	228	118	167	251	134	183	242	131	178
**2011**	188	92	133	182	88	129	209	102	148	225	117	163	213	96	145
**2013**	177	86	126	172	83	122	201	97	143	206	108	151	187	95	134

Notes: Directly age-standardised to the European Standard Population 2013.

Source: 1969 to 2009: England and Wales, Office for National Statistics (2010) Personal communication.
Scotland; General Register Office (2010) Personal communication.
Northern Ireland, Statistics and Research Agency (2010) Personal communication.
2010 to 2013: England and Wales, Office for National Statistics; Scotland, General Register Office for Scotland; Northern Ireland, Northern Ireland Statistics and Research Agency.

Age-standardised death rates per 100,000 from stroke, all ages, United Kingdom and England, Wales, Scotland, Northern Ireland, 1979 to 2013

	United Kingdom			England			Wales			Scotland			Northern Ireland		
**Year**	**Men**	**Women**	**Both**	**Men**	**Women**	**Both**	**Men**	**Women**	**Both**	**Men**	**Women**	**Both**	**Men**	**Women**	**Both**
**1979**	254	232	242	243	223	232	274	256	266	335	298	313	341	277	300
**1981**	229	210	219	220	201	210	245	233	241	308	273	287	240	253	252
**1983**	213	199	206	206	191	199	228	214	221	272	255	264	235	238	238
**1985**	224	204	213	219	199	208	228	220	225	272	240	253	223	189	201
**1987**	203	189	196	198	184	191	209	199	205	255	229	240	202	192	198
**1989**	195	179	187	190	174	181	194	187	192	253	228	239	198	184	191
**1991**	192	174	182	189	170	179	194	175	184	232	211	221	172	172	174
**1993**	167	156	162	160	149	154	161	156	161	241	222	231	201	165	180
**1995**	156	147	152	151	141	146	165	153	159	212	201	208	167	158	163
**1997**	149	139	144	144	135	140	153	142	148	196	174	184	167	148	156
**1999**	139	134	137	134	130	133	141	136	140	184	171	177	162	154	157
**2001**	144	134	139	140	130	136	149	141	145	174	162	168	148	136	142
**2003**	139	131	135	135	128	132	153	135	143	170	159	165	142	133	138
**2005**	115	112	115	113	110	112	118	113	117	142	139	142	114	109	112
**2007**	101	99	101	98	96	98	107	102	105	129	121	125	109	108	109
**2009**	91	89	91	89	87	88	95	97	97	110	110	111	91	92	93
**2011**	76	73	75	73	70	72	75	75	76	100	98	100	80	82	82
**2013**	70	68	69	67	65	66	79	71	74	96	92	95	79	75	77

Notes: Directly age-standardised to the European Standard Population 2013.

Source: 1969 to 2009: England and Wales, Office for National Statistics (2010) Personal communication.
Scotland; General Register Office (2010) Personal communication.
Northern Ireland, Statistics and Research Agency (2010) Personal communication.
2010 to 2013: England and Wales, Office for National Statistics; Scotland, General Register Office for Scotland; Northern Ireland, Northern Ireland Statistics and Research Agency.

Age-standardised death rates per 100,000 from cardiovascular disease (CVD), under 75, United Kingdom and England, Wales, Scotland, Northern Ireland, 1979 to 2013

	United Kingdom			England			Wales			Scotland			Northern Ireland		
**Year**															
**1979**	462	211	325	447	201	314	504	230	354	545	275	395	583	279	415
**1981**	432	196	304	418	187	293	469	210	328	513	259	373	534	245	375
**1983**	417	188	293	404	179	282	464	206	324	498	246	359	495	242	356
**1985**	397	179	279	384	170	269	433	194	304	480	241	348	473	228	339
**1987**	369	168	261	358	160	251	390	181	278	453	224	327	424	200	301
**1989**	342	158	243	329	150	233	363	171	260	433	217	314	404	185	284
**1991**	319	147	227	309	141	219	337	150	237	392	196	285	353	168	252
**1993**	304	140	217	293	134	209	329	149	233	381	186	275	346	161	245
**1995**	274	127	196	265	121	189	294	137	210	336	165	243	315	155	228
**1997**	248	117	179	239	112	172	271	129	196	307	153	224	282	129	199
**1999**	225	105	162	217	101	156	251	113	178	280	136	202	250	114	177
**2001**	199	93	144	194	90	140	219	105	159	240	117	174	201	97	145
**2003**	180	84	130	175	81	126	200	93	144	223	106	160	181	79	127
**2005**	155	71	111	150	68	108	172	80	124	193	91	139	156	73	112
**2007**	137	62	98	132	60	95	146	70	107	179	82	128	141	63	100
**2009**	122	54	87	118	52	84	134	63	97	148	71	108	122	60	90
**2011**	108	47	76	104	45	73	117	56	86	137	63	98	114	46	79
**2013**	101	45	72	98	43	70	113	51	81	127	60	92	99	45	71

Notes: Directly age-standardised to the European Standard Population 2013.

Source: 1969 to 2009: England and Wales, Office for National Statistics (2010) Personal communication.
Scotland; General Register Office (2010) Personal communication.
Northern Ireland, Statistics and Research Agency (2010) Personal communication.
2010 to 2013: England and Wales, Office for National Statistics; Scotland, General Register Office for Scotland; Northern Ireland, Northern Ireland Statistics and Research Agency.

Age-standardised death rates per 100,000 from coronary heart disease (CHD), under 75,United Kingdom and England, Wales, Scotland, Northern Ireland, 1979 to 2013

	United Kingdom			England			Wales			Scotland			Northern Ireland		
**Year**	**Men**	**Women**	**Both**	**Men**	**Women**	**Both**	**Men**	**Women**	**Both**	**Men**	**Women**	**Both**	**Men**	**Women**	**Both**
**1979**	319	111	206	308	104	198	345	120	222	384	149	254	403	155	267
**1981**	304	107	197	294	101	190	324	111	208	368	148	247	388	147	256
**1983**	298	105	194	287	99	186	332	111	212	365	146	244	366	138	241
**1985**	290	105	190	280	99	183	319	113	208	353	147	239	347	135	231
**1987**	271	100	179	261	95	172	288	108	192	338	140	229	319	124	212
**1989**	247	95	165	238	89	158	266	101	178	314	133	214	298	115	198
**1991**	230	88	154	222	84	149	246	90	163	284	120	194	265	102	176
**1993**	217	81	145	208	77	139	237	89	158	274	112	186	254	102	171
**1995**	190	71	127	183	67	121	209	74	137	236	96	160	232	95	157
**1997**	168	63	113	162	60	108	184	69	124	210	86	143	204	75	134
**1999**	151	55	100	144	52	96	171	58	112	193	75	130	178	64	117
**2001**	131	48	87	127	45	84	148	55	99	159	63	108	139	54	94
**2003**	116	41	77	112	39	74	127	46	85	149	56	99	119	41	77
**2005**	99	34	65	95	31	62	111	38	73	130	48	86	106	39	71
**2007**	86	28	56	82	27	53	90	32	60	117	42	77	97	32	63
**2009**	75	24	48	72	22	46	84	27	55	95	35	63	81	30	54
**2011**	66	21	43	63	19	41	71	24	47	87	29	56	75	22	48
**2013**	62	19	40	60	18	38	72	23	47	80	26	52	65	20	42

Notes: Directly age-standardised to the European Standard Population 2013.

Source: 1969 to 2009: England and Wales, Office for National Statistics (2010) Personal communication.
Scotland; General Register Office (2010) Personal communication.
Northern Ireland, Statistics and Research Agency (2010) Personal communication.
2010 to 2013: England and Wales, Office for National Statistics; Scotland, General Register Office for Scotland; Northern Ireland, Northern Ireland Statistics and Research Agency.

Age-standardised death rates per 100,000 from stroke, under 75, United Kingdom and England, Wales, Scotland, Northern Ireland, 1979 to 2013

	United Kingdom			England			Wales			Scotland			Northern Ireland		
**Year**	**Men**	**Women**	**Both**	**Men**	**Women**	**Both**	**Men**	**Women**	**Both**	**Men**	**Women**	**Both**	**Men**	**Women**	**Both**
**1979**	76	57	66	72	54	62	85	64	73	102	79	89	96	74	84
**1981**	67	51	58	64	48	55	76	57	65	92	73	81	76	59	67
**1983**	62	48	54	60	45	52	68	53	60	83	66	73	72	69	71
**1985**	61	46	52	58	44	50	65	51	58	81	62	70	69	54	60
**1987**	55	42	48	53	40	46	56	45	50	73	54	62	58	43	50
**1989**	51	39	45	49	37	42	51	44	47	72	55	63	60	43	51
**1991**	49	36	42	47	34	40	53	36	44	65	48	55	48	42	45
**1993**	42	32	37	40	30	35	41	32	36	62	47	54	46	33	39
**1995**	40	30	35	38	29	33	40	31	35	58	42	49	42	35	38
**1997**	37	28	32	36	27	31	41	30	35	49	36	42	43	30	36
**1999**	34	26	30	33	25	29	37	27	31	43	34	38	40	29	34
**2001**	32	24	28	31	23	27	31	25	28	40	31	35	28	23	25
**2003**	29	22	26	28	21	25	32	25	28	38	28	32	30	19	24
**2005**	24	19	21	24	18	21	26	20	23	30	24	27	24	16	20
**2007**	21	16	18	20	15	18	23	18	21	29	20	24	19	18	18
**2009**	18	14	16	18	13	15	18	16	17	25	20	22	18	14	16
**2011**	17	12	14	16	12	14	18	15	16	24	16	20	18	13	15
**2013**	15	11	13	15	11	12	16	11	14	21	17	19	14	12	13

Notes: Directly age-standardised to the European Standard Population 2013.

Source: 1969 to 2009: England and Wales, Office for National Statistics (2010) Personal communication.
Scotland; General Register Office (2010) Personal communication.
Northern Ireland, Statistics and Research Agency (2010) Personal communication.
2010 to 2013: England and Wales, Office for National Statistics; Scotland, General Register Office for Scotland; Northern Ireland, Northern Ireland Statistics and Research Agency.

Prevalence of cardiovascular conditions in men by age, Great Britain 1988-2011

		1988	1989	1994	1995	1996	1998	2000	2001	2002	2003	2004	2005	2006	2007	2008	2009	2010	2011
		%	%	%	%	%	%	%	%	%	%	%	%	%	%	%	%	%	%
Myocardial infarction	16-44	0.2	0.2	0.1	0.1	0.1	0.3	0.1	0.1	0.1	0.2	0.0	0.1	0.2	0.0	*	0.2	0.2	0.1
	45-64	4.7	4.6	3.1	3.4	3.0	4.0	3.1	3.2	2.6	2.2	2.6	3.0	2.4	2.2	2.2	2.1	2.1	1.9
	65-74	7.5	10.0	6.6	9.2	6.6	8.9	8.6	7.5	7.0	8.7	8.4	6.2	6.4	6.5	5.4	5.8	5.8	4.7
	75+	8.2	7.9	8.1	5.4	7.5	11.1	9.4	11.3	11.8	8.0	8.3	6.5	7.2	5.7	5.8	6.3	6.3	7.2
	**All Ages**	**2.2**	**2.3**	**2.3**	**2.5**	**2.3**	**3.2**	**2.5**	**2.5**	**2.4**	**2.3**	**2.3**	**2.1**	**2.0**	**1.8**	**1.7**	**1.9**	**1.9**	**1.7**
Stroke	16-44	0.0	0.0	0.1	0.0	0.1	0.1	0.1	0.2	0.1	0.1	0.1	0.1	0.1	0.0	0.1	*	*	0.1
	45-64	1.0	1.0	1.0	0.6	0.9	0.8	1.1	1.1	0.8	0.9	0.8	1.0	0.7	0.8	0.6	0.8	0.8	0.8
	65-74	3.9	3.0	3.0	2.0	2.7	2.6	3.6	1.8	2.6	2.0	1.7	1.8	3.2	2.8	2.4	2.3	2.3	1.7
	75+	3.8	4.8	3.7	3.1	4.3	3.0	3.6	2.3	3.9	3.7	5.4	3.5	3.5	3.8	3.3	3.1	3.1	2.6
	**All Ages**	**0.8**	**0.7**	**0.9**	**0.6**	**1.0**	**0.8**	**1.0**	**0.8**	**0.9**	**0.8**	**0.8**	**0.8**	**0.9**	**0.8**	**0.7**	**0.7**	**0.7**	**0.7**
CVD	16-44	1.7	1.2	1.6	1.2	1.4	1.9	1.8	2.2	1.7	1.7	1.4	1.5	1.3	1.0	1.4	1.6	1.5	1.5
	45-64	14.3	13.3	13.8	12.9	14.1	15.5	13.7	15.0	15.2	14.7	14.6	16.7	15.9	14.7	15.6	14.9	14.6	14.6
	65-74	24.7	25.9	24.6	27.2	26.8	28.1	29.0	31.3	33.0	34.5	29.5	28.8	35.5	32.0	31.2	32.2	33.9	28.5
	75+	22.3	22.1	23.6	23.8	24.9	31.0	30.8	33.3	39.8	31.7	37.3	32.9	38.4	33.8	31.1	33.7	35.5	34.1
	**All Ages**	**7.3**	**6.9**	**9.3**	**9.3**	**9.9**	**11.3**	**10.7**	**11.0**	**11.9**	**11.3**	**11.1**	**11.4**	**12.0**	**10.9**	**11.1**	**11.4**	**11.7**	**11.1**

*Notes: * Information is suppressed for low cell count as a measure of disclosure control.*

Source: Office for National Statistics (2006) 2005 General Household Survey.
Office for National Statistics (2007-2012) General Lifestyle Survey 2006-2011.

Prevalence of cardiovascular conditions in women by age, Great Britain 1988-2011

		1988	1989	1994	1995	1996	1998	2000	2001	2002	2003	2004	2005	2006	2007	2008	2009	2010	2011
		%	%	%	%	%	%	%	%	%	%	%	%	%	%	%	%	%	%
Myocardial infarction	16-44	0.1	0.1	0.1	0.1	0.1	0.0	0.1	0.0	0.0	0.1	0.0	0.0	0.1	0.1	*	0.1	0.1	0.1
	45-64	2.0	2.4	2.0	1.7	2.8	1.8	1.8	1.6	1.5	1.3	1.8	1.4	1.2	0.8	0.8	0.9	0.7	0.8
	65-74	6.7	6.4	5.9	4.5	6.5	6.5	5.4	3.9	5.8	6.3	5.1	4.8	3.9	2.6	2.6	2.9	3.1	3.1
	75+	6.6	6.5	7.3	7.3	8.3	8.6	7.8	5.3	9.0	5.2	6.7	7.0	6.5	5.1	4.5	4.9	4.6	3.7
	**All Ages**	**1.7**	**1.7**	**2.1**	**1.8**	**2.4**	**2.2**	**2.0**	**1.5**	**2.0**	**1.7**	**1.8**	**1.7**	**1.5**	**1.1**	**1.0**	**1.2**	**1.1**	**1.0**
Stroke	16-44	0.1	0.1	0.2	0.0	0.0	0.1	0.1	0.0	0.1	0.0	0.0	0.1	0.1	0.1	*	0.1	0.1	0.1
	45-64	0.4	0.4	0.5	0.5	0.5	0.5	0.7	0.7	0.8	0.4	0.5	0.4	0.5	0.5	0.6	0.4	0.7	0.3
	65-74	2.4	1.5	1.8	1.9	1.7	1.5	1.7	2.0	2.2	1.9	1.2	1.5	1.7	1.2	1.6	1.3	1.4	1.2
	75+	3.0	4.0	3.6	3.5	2.2	4.1	3.3	3.9	3.7	2.6	2.8	3.0	2.1	1.9	2.7	2.1	1.8	1.4
	**All Ages**	**0.6**	**0.6**	**0.8**	**0.7**	**0.6**	**0.8**	**0.8**	**0.9**	**0.9**	**0.6**	**0.6**	**0.6**	**0.6**	**0.5**	**0.6**	**0.5**	**0.6**	**0.4**
CVD	16-44	1.7	2.2	1.7	1.3	1.5	1.3	1.8	1.5	1.9	2.2	1.7	2.0	2.3	1.8	1.2	1.7	2.1	1.9
	45-64	10.8	11.5	10.6	9.7	12.4	10.6	11.7	11.5	12.9	11.8	13.2	12.3	12.8	11.0	9.9	9.8	10.6	8.4
	65-74	22.8	22.0	23.9	19.7	22.4	26.8	26.2	25.2	29.1	29.7	26.6	26.0	29.0	23.9	23.0	26.2	22.3	22.5
	75+	26.5	26.8	25.1	29.2	25.4	29.9	30.6	32.2	37.9	30.3	31.9	31.3	33.3	27.7	32.5	27.5	31.7	29.8
	**All Ages**	**7.7**	**7.7**	**9.2**	**8.7**	**9.5**	**9.9**	**10.4**	**10.2**	**11.9**	**10.9**	**11.0**	**10.8**	**11.5**	**9.7**	**9.4**	**9.5**	**10.1**	**9.1**

*Notes: * Information is suppressed for low cell count as a measure of disclosure control.*

Source: Office for National Statistics (2006) 2005 General Household Survey.
Office for National Statistics (2007-2012) General Lifestyle Survey 2006-2011.

Prevalence of selected heart conditions by gender, from the health surveys of England, Scotland and Wales 2003 to 2014

		2003	2006	2007	2008	2009	2010	2011	2012	2013	2014
		%		%	%	%	%	%	%	%	%
**Coronary Heart Disease**											
England	Men	6.4	6.5					5.7			
	Women	4.1	4.0					3.5			
	**All adults**	**5.2**	**5.2**					**4.6**			
Scotland	Men	8.2			6.9	7.4	7.5	7.5	7.3	7.1	
	Women	6.5			5.6	5.2	5.2	4.9	5.7	5.3	
	**All adults**	**7.3**			**6.2**	**6.2**	**6.3**	**6.2**	**6.5**	**6.1**	
**Myocardial infarction**											
England	Men	3.8	4.1					3.7			
	Women	1.7	1.7					1.6			
	**All adults**	**-**	**2.9**					**2.6**			
Wales	Men	6.0		6.0	6.0	5.0	5.0	5.1	5.1	5.0	4.6
	Women	3.0		3.0	2.0	3.0	2.6	2.5	2.6	2.3	2.4
	**All adults**	**5.0**		**4.0**	**4.0**	**4.0**	**3.8**	**3.8**	**3.8**	**3.6**	**3.5**
**Stroke**											
England	Men	2.4	2.4					2.7			
	Women	2.2	2.2					2.1			
	**All adults**	**2.3**	**2.3**					**2.4**			
Scotland	Men	2.4			2.5	2.7	3.3	2.9	2.8	3.2	
	Women	2.1			2.8	2.2	2.5	2.7	2.8	2.7	
	**All adults**	**2.2**			**2.6**	**2.5**	**2.9**	**2.8**	**2.8**	**2.9**	

Cardiovascular Diseases

England	Men	13.6	13.6					13.9			
	Women	13.0	13.0					13.4			
	**All adults**		**13.3**					**13.6**			
Scotland	Men	14.9			15.1	15.2	16.3	15.6	16.6	15.7	
	Women	14.5			15.5	13.7	14.0	13.8	15.9	15.3	
	**All adults**	**14.7**			**15.3**	**14.4**	**15.1**	**14.6**	**16.2**	**15.5**	
**Any heart condition**											
Wales	Men	11.0		11.0	10.0	10.0	9.5	9.7	9.9	9.9	9.6
	Women	9.0		8.0	8.0	8.0	7.3	7.6	7.6	7.0	7.5
	**All adults**	**10.0**		**9.0**	**9.0**	**9.0**	**8.4**	**8.6**	**8.7**	**8.4**	**8.5**

Notes: Adults aged 16 years and over.
England - Data have been weighted for non-response. *Copyright © 2014, Re-used with the permission of The Health and Social Care Information Centre. All rights reserved.*

Source: Health & Social Care Information Centre. (2014). Health Survey for England 2013, Trend Tables. www.hscic.gov.uk (accessed June 2015).
The Scottish Government (2014). The Scottish Health Survey 2013. http://www.gov.scot/Publications/2014/12/9982 (accessed June 2015).
The Welsh Government. Welsh Health Surveys: 2003/04-2014. http://gov.wales/statistics-and-research/welsh-health-survey/?tab=previous&lang=en (accessed September 2015).

Inpatient episodes by main diagnosis in National Health Service hospitals in men, England, Scotland, Wales, Northern Ireland and United Kingdom, 2005/06-2013/14

		2005/06	2006/07	2007/08	2008/09	2009/10	2010/11	2011/12	2012/13	2013/14
England	All diseases of the circulatory system (CVD) (I00-I99)	694,974	705,822	715,200	741,384	759,672	767,889	779,921	777,888	793,952
	Coronary heart disease (I20-I25)	274,816	276,900	275,069	274,163	265,667	263,538	266,954	265,102	264,934
	Stroke (I60-I69)	85,041	84,271	86,372	91,363	97,878	96,364	95,294	96,502	97,593
	Other cardiovascular disease	335,117	344,651	353,759	375,858	396,127	407,987	417,673	416,284	431,425
Scotland	All diseases of the circulatory system (CVD) (I00-I99)					80,575	83,336	83,040	83,723	87,460
	Coronary heart disease (I20-I25)					30,871	32,357	31,154	31,154	31,695
	Stroke (I60-I69)					10,898	10,965	10,864	11,565	9,430
	Other cardiovascular disease					38,806	40,014	41,022	41,004	46,335
Wales	All diseases of the circulatory system (CVD) (I00-I99)	40,598	41,128	41,525	43,120	44,510	44,492	42,800	45,446	49,671
	Coronary heart disease (I20-I25)	15,153	15,597	15,821	15,946	15,915	15,621	15,240	15,745	16,806
	Stroke (I60-I69)	5,461	5,460	5,304	5,715	5,827	6,045	5,389	5,672	6,617
	Other cardiovascular disease	19,984	20,071	20,400	21,459	22,768	22,826	22,171	24,029	26,248
Northern Ireland	All diseases of the circulatory system (CVD) (I00-I99)						24,499	26,591	24,154	25,674
	Coronary heart disease (I20-I25)						9,800	9,521	9,846	10,697
	Stroke (I60-I69)						2,485	1,628	1,437	1,742
	Other cardiovascular disease						12,214	15,442	12,871	13,235
**United Kingdom**	**All diseases of the circulatory system (CVD) (I00-I99)**						**920,216**	**932,352**	**931,211**	**956,757**
	Coronary heart disease (I20-I25)						321,316	322,869	321,847	324,132
	Stroke (I60-I69)						115,859	113,175	115,176	115,382
	Other cardiovascular disease						483,041	496,308	494,188	517,243

Inpatient episodes by main diagnosis in National Health Service hospitals in women, England, Scotland, Wales, Northern Ireland and United Kingdom, 2005/06-2013/14

		2005/06	2006/07	2007/08	2008/09	2009/10	2010/11	2011/12	2012/13	2013/14
England	All diseases of the circulatory system (CVD) (I00-I99)	549,030	549,768	559,474	580,911	598,575	603,920	601,714	596,206	607,280
	Coronary heart disease (I20-I25)	153,446	151,013	149,178	148,171	142,008	141,558	142,554	138,987	136,073
	Stroke (I60-I69)	93,280	92,181	93,627	98,738	105,827	101,971	99,142	99,579	99,763
	Other cardiovascular disease	302,304	306,574	316,669	334,002	350,740	360,391	360,018	357,640	371,444
Scotland	All diseases of the circulatory system (CVD) (I00-I99)					64,325	65,696	65,699	65,043	69,838
	Coronary heart disease (I20-I25)					17,052	17,800	17,545	16,486	17,920
	Stroke (I60-I69)					11,543	11,674	11,798	12,172	9,827
	Other cardiovascular disease					35,730	36,222	36,356	36,385	42,091
Wales	All diseases of the circulatory system (CVD) (I00-I99)	34,220	34,207	34,456	35,594	37,089	36,410	34,789	35,507	38,434
	Coronary heart disease (I20-I25)	9,357	9,461	9,335	9,128	8,978	8,679	8,664	8,590	8,841
	Stroke (I60-I69)	6,199	5,683	5,884	6,598	6,584	6,426	6,071	6,408	6,648
	Other cardiovascular disease	18,664	19,063	19,237	19,868	21,527	21,305	20,054	20,509	22,945
Northern Ireland	All diseases of the circulatory system (CVD) (I00-I99)						18,388	20,026	18,721	18,622
	Coronary heart disease (I20-I25)						4,609	4,539	4,674	4,681
	Stroke (I60-I69)						2,387	1,571	1,506	1,641
	Other cardiovascular disease						11,392	13,916	12,541	12,300
**United Kingdom**	**All diseases of the circulatory system (CVD) (I00-I99)**						**724,414**	**722,228**	**715,477**	**734,174**
	Coronary heart disease (I20-I25)						172,646	173,302	168,737	167,515
	Stroke (I60-I69)						122,458	118,582	119,665	117,879
	Other cardiovascular disease						429,310	430,344	427,075	448,780

Notes: Finished consultant episodes; ordinary admissions and day cases combined. Pregnancy cases not included. ICD-10 codes in parentheses.

Source: Department of Health (2014). Hospital Episode Statistics 2013/14. www.hesonline.nhs.uk (accessed April 2015).

Information Services Division Scotland (2014) Main diagnosis discharges from hospital 2013/14. www.isdscotland.org (accessed April 2015). Personal correspondence.

NHS Wales Informatics Service (2014). The Patient Episode Database for Wales- 2013/14. www.infoandstats.wales.nhs.uk (accessed April 2015). Hospital Information Branch (2014). Northern Ireland Episode Based Acute Inpatient and Day Case Activity Data (2013/14) www.dhsspsni.gov.uk (accessed April 2015). Personal correspondence.

Inpatient episodes by main diagnosis in National Health Service hospitals in men, England 2000/01 to 2013/14

CVD	0 to 4	5 to 9	10 to 14	15 to 19	20 to 24	25 to 29	30 to 34	35 to 39	40 to 44	45 to 49	50 to 54	55 to 59	60 to 64	65 to 69	70 to 74	75 to 79	80 to 84	85 to 89	90+	All ages
**2000/01**	1,056	1,313	974	1,573	2,366	4,193	7,133	11,114	16,383	24,024	38,272	46,408	57,723	64,613	65,150	58,654	36,360	20,430	7,219	**464,958**
**2001/02**	1,077	1,328	927	1,577	2,434	4,231	7,130	11,128	16,677	23,815	37,220	47,499	56,029	63,518	64,915	57,621	38,059	20,782	7,691	**463,658**
**2002/03**	1,036	1,313	944	1,785	2,734	4,340	7,558	11,699	17,600	24,987	36,661	50,668	56,328	63,887	66,537	59,370	42,101	21,158	8,614	**479,320**
**2003/04**	1,184	1,312	1,003	1,907	3,142	4,592	7,752	12,128	18,395	25,615	36,247	51,442	57,145	64,457	66,929	60,188	44,532	20,670	9,201	**487,841**
**2004/05**	1,129	1,219	921	1,916	3,182	4,378	7,399	12,266	18,552	26,484	35,853	52,007	57,535	65,363	67,623	60,113	46,503	20,655	9,470	**492,568**
**2005/06**	1,235	1,353	939	2,086	3,353	4,708	8,097	12,529	20,018	26,933	35,967	53,710	59,382	65,948	69,595	62,708	46,510	23,446	10,071	**508,588**
**2006/07**	1,245	1,363	1,067	1,955	3,471	4,751	7,766	12,895	19,822	27,972	37,524	53,978	61,343	64,778	70,136	63,123	46,468	24,605	9,890	**514,152**
**2007/08**	1,357	1,397	997	2,073	3,229	5,073	7,224	12,830	20,332	28,311	36,950	50,506	64,330	64,538	69,357	64,133	46,533	26,248	10,141	**515,559**
**2008/09**	1,496	1,449	1,114	2,339	3,444	5,513	7,529	12,833	20,060	29,154	37,481	50,241	65,196	64,790	70,835	65,185	48,396	29,040	10,520	**526,615**
**2009/10**	1,560	1,470	1,015	2,299	3,513	5,629	7,568	12,379	19,836	29,198	37,563	49,028	64,901	65,298	71,000	65,643	49,907	30,608	10,806	**529,221**
**2010/11**	1,520	1,435	1,099	2,210	3,448	5,363	7,558	11,985	19,927	29,184	37,794	48,632	64,869	65,978	70,263	67,012	51,166	31,124	12,318	**532,885**
**2011/12**	1,716	1,419	1,213	2,224	3,565	5,161	7,477	11,181	19,242	29,096	39,052	48,663	64,350	68,409	69,683	67,498	52,465	32,165	13,970	**538,549**
**2012/13**	1,620	1,412	1,386	2,266	3,623	5,165	7,602	10,937	18,916	29,003	39,706	47,675	60,346	70,447	66,183	66,269	53,437	32,054	14,385	**532,432**
**2013/14**	1,794	1,556	1,387	2,349	3,610	5,238	8,075	10,548	18,712	29,026	40,409	49,372	59,382	72,446	67,030	66,779	53,862	32,398	15,039	**539,012**
**CHD**																				
**2000/01**	13	7	5	18	49	154	668	2,479	6,164	11,223	19,568	24,289	29,574	30,812	27,297	20,750	11,048	5,343	1,491	**190,952**
**2001/02**	14	1	3	16	44	185	691	2,370	6,192	11,148	19,136	24,916	29,041	30,529	28,301	21,118	11,949	5,464	1,756	**192,874**
**2002/03**	7	4	2	19	48	173	752	2,446	6,351	11,587	18,600	26,321	28,850	31,075	29,408	22,630	13,584	5,941	2,065	**199,863**
**2003/04**	15	6	2	20	72	187	760	2,466	6,474	12,015	18,238	26,297	29,146	31,413	29,565	22,904	14,463	5,795	2,351	**202,189**
**2004/05**	13	1	2	29	80	172	706	2,448	6,431	12,036	18,064	26,587	29,295	31,467	30,253	23,325	15,104	5,870	2,318	**204,201**

**2005/06**	24	2	3	29	76	215	753	2,432	6,647	11,645	17,377	26,504	29,370	30,926	29,818	24,159	14,697	6,752	2,497	**203,926**
**2006/07**	13	5	1	28	76	192	745	2,451	6,352	11,962	17,751	26,402	29,814	30,222	30,118	24,405	14,860	7,031	2,467	**204,895**
**2007/08**	9	3	3	32	107	228	688	2,423	6,346	11,773	17,278	24,708	30,856	29,607	29,575	24,924	15,260	7,271	2,443	**203,534**
**2008/09**	16	6	13	22	89	198	648	2,204	5,993	11,418	16,867	23,977	30,373	28,511	29,613	24,923	15,259	7,828	2,547	**200,505**
**2009/10**	9	7	5	22	77	195	601	2,082	5,631	11,169	16,615	21,933	28,629	27,436	28,302	23,485	15,003	7,943	2,570	**191,714**
**2010/11**	17	7	8	25	77	169	555	1,908	5,429	10,750	16,101	21,179	27,945	26,727	27,224	23,373	15,354	7,745	2,886	**187,479**
**2011/12**	21	8	4	25	75	207	531	1,746	5,251	10,655	16,462	21,319	27,418	27,509	26,383	23,534	15,825	8,037	3,240	**188,250**
**2012/13**	20	7	10	36	100	155	516	1,650	5,038	10,434	16,545	21,159	25,875	28,547	25,542	23,380	16,327	8,135	3,190	**186,666**
**2013/14**	22	3	18	25	87	172	618	1,469	4,774	10,299	16,687	21,422	25,433	28,908	25,346	23,212	15,970	7,842	3,112	**185,419**
**Stroke**																				
**2000/01**	87	45	54	88	117	192	335	542	786	1,323	2,159	2,915	4,002	5,705	7,330	8,343	6,274	4,151	1,585	**46,033**
**2001/02**	86	60	35	107	139	247	374	619	874	1,303	2,238	3,047	3,952	5,565	7,108	8,293	6,716	4,287	1,688	**46,738**
**2002/03**	111	52	49	156	120	190	360	603	905	1,409	2,126	3,222	4,078	5,619	7,191	8,149	7,329	4,276	1,900	**47,845**
**2003/04**	113	41	57	116	156	193	332	633	1,026	1,370	2,151	3,215	4,084	5,546	6,854	8,025	7,479	4,137	1,943	**47,471**
**2004/05**	119	56	67	105	136	174	319	656	1,006	1,606	2,411	3,153	4,267	5,555	6,814	7,720	7,476	3,846	1,938	**47,424**
**2005/06**	129	70	43	115	121	212	356	660	1,058	1,543	2,081	3,374	4,221	5,444	6,916	7,906	7,460	4,156	2,108	**47,973**
**2006/07**	115	48	72	98	142	224	374	646	1,084	1,575	2,195	3,236	4,138	5,191	6,394	7,479	7,082	4,415	2,073	**46,581**
**2007/08**	138	53	57	97	147	236	290	658	1,059	1,579	2,114	3,019	4,363	5,223	6,545	7,324	7,006	4,544	2,085	**46,537**
**2008/09**	152	49	51	93	145	241	322	600	1,136	1,746	2,238	3,064	4,431	5,263	6,681	7,684	7,043	5,168	2,102	**48,209**
**2009/10**	128	66	60	113	158	211	329	631	1,202	1,806	2,414	3,351	4,842	5,629	6,951	8,047	7,569	5,256	2,115	**50,878**
**2010/11**	135	45	49	112	146	195	349	691	1,192	1,872	2,607	3,550	4,922	5,860	7,122	8,286	7,644	5,707	2,521	**53,005**
**2011/12**	124	61	65	111	143	237	364	669	1,151	1,965	2,755	3,687	4,929	6,067	7,208	8,199	7,883	5,664	2,761	**54,043**
**2012/13**	130	46	104	82	137	219	332	587	1,160	2,012	2,909	3,616	4,884	6,294	7,232	8,206	8,120	5,631	2,811	**54,512**

**2013/ 14**	164	61	56	96	146	224	408	612	1,202	1,978	3,016	3,683	4,601	6,337	7,135	8,012	7,931	5,615	2,888	**54,165**

Notes: Hospital Episode Statistics (HES) include activity ending in the year in question and run from April to March, e.g. 2012-13 includes activity ending between 1st April 2012 and 31st March 2013. A finished admission episode (FAE) is the first period of admitted patient care under one consultant within one healthcare provider. Admissions do not represent the number of patients, as a person may have more than one admission within the period. FAEs are counted against the year or month in which the admission episode finishes.

Source: HSCIC (2015). Hospital Episode Statistics (HES). Health and Social Care Information Centre. Leeds.

Inpatient episodes by main diagnosis in National Health Service hospitals in women, England 2000/01 to 2013/14

CVD	**0 to 4**	**5 to 9**	**10 to 14**	**15 to 19**	**20 to 24**	**25 to 29**	**30 to 34**	**35 to 39**	**40 to 44**	**45 to 49**	**50 to 54**	**55 to 59**	**60 to 64**	**65 to 69**	**70 to 74**	**75 to 79**	**80 to 84**	**85 to 89**	**90+**	**All ages**
**2000/01**	771	854	1,020	1,304	2,494	5,067	8,388	10,489	11,839	14,777	21,223	24,101	29,978	37,783	45,976	53,114	45,046	36,543	19,900	**370,667**
**2001/02**	784	784	1,118	1,295	2,472	4,491	7,853	10,239	11,885	14,474	20,033	24,246	28,616	36,139	44,794	51,018	46,355	35,629	20,777	**363,002**
**2002/03**	826	853	1,194	1,285	2,738	4,628	8,171	11,230	13,168	15,490	20,231	25,954	29,464	36,774	46,039	51,307	49,710	36,275	22,792	**378,129**
**2003/04**	867	831	1,175	1,495	3,262	4,594	7,825	11,087	13,270	15,538	19,541	26,576	29,823	37,849	45,869	51,355	52,020	34,484	23,380	**380,841**
**2004/05**	938	754	1,158	1,384	3,031	4,545	7,588	10,538	13,276	15,222	18,947	25,855	29,451	38,323	45,363	51,513	53,336	33,709	24,359	**379,290**
**2005/06**	917	888	1,116	1,716	3,155	4,872	7,489	11,086	14,373	16,152	19,375	26,662	30,216	38,753	46,444	52,377	51,585	36,488	24,995	**388,659**
**2006/07**	1,035	816	1,087	1,679	3,214	5,121	7,283	11,294	14,380	16,608	19,997	26,924	30,991	37,960	45,894	52,847	49,221	36,760	23,968	**387,079**
**2007/08**	1,089	854	1,127	1,749	3,288	5,278	7,169	10,753	14,430	17,582	20,143	25,698	33,489	37,352	45,536	52,032	48,603	38,745	24,389	**389,306**
**2008/09**	2,282	1,707	2,437	3,705	6,986	10,973	14,164	21,978	29,855	36,734	40,461	49,313	66,958	75,079	92,383	105,056	99,826	84,999	48,447	**793,343**
**2009/10**	-	-	-	-	-	-	-	-	-	-	-	-	-	-	-	-	-	-	-	**-**
**2010/11**	1,152	981	1,248	1,872	3,569	5,458	7,052	10,326	14,531	18,648	20,854	24,553	33,092	37,859	45,829	52,659	50,653	42,343	27,107	**399,786**
**2011/12**	1,117	971	1,288	1,795	3,486	5,175	6,787	9,266	13,704	17,789	20,815	24,026	32,690	38,055	45,587	51,654	52,214	42,266	28,432	**397,117**
**2012/13**	1,265	1,110	1,315	1,901	3,422	5,091	6,662	8,321	12,906	17,484	20,583	23,340	29,993	38,856	43,780	50,395	51,283	41,421	29,875	**389,003**
**2013/14**	1,368	1,141	1,379	1,879	3,520	5,211	6,965	8,670	12,732	17,443	21,621	24,529	29,893	40,994	43,839	51,191	51,590	41,027	29,764	**394,756**
**CHD**																				
**2000/01**	5	2	4	3	25	34	193	595	1,670	3,322	6,084	8,085	11,635	14,586	16,245	15,657	12,062	8,273	3,812	**102,292**
**2001/02**	3	2	1	9	36	61	218	631	1,704	3,256	5,683	8,287	11,050	14,124	16,398	15,477	12,470	8,406	4,055	**101,871**

**2002/03**	6	1	2	5	14	62	241	686	1,851	3,487	5,645	8,660	11,200	14,308	16,874	16,154	13,523	8,663	4,853	**106,235**
**2003/04**	4	2	2	12	20	82	217	742	1,910	3,471	5,722	8,745	10,871	14,660	17,025	16,490	14,260	8,323	5,163	**107,721**
**2004/05**	7	2	1	3	27	64	242	630	1,908	3,506	5,437	8,470	10,959	14,723	16,707	16,742	14,412	8,114	5,232	**107,186**
**2005/06**	5	5	1	24	29	60	197	654	1,967	3,772	5,570	8,743	10,831	14,476	16,765	17,085	13,934	8,701	5,350	**108,169**
**2006/07**	4	4	-	20	23	58	232	731	1,934	3,796	5,608	8,671	10,803	13,861	16,272	16,730	13,158	8,548	5,203	**105,656**
**2007/08**	13	6	1	15	27	69	214	658	1,923	3,773	5,794	7,917	11,353	13,379	15,659	16,515	12,875	9,062	5,165	**104,418**
**2008/09**	17	5	7	19	59	147	330	1,336	3,462	7,259	10,722	14,213	20,927	24,384	29,700	30,974	25,329	18,369	9,926	**197,185**
**2009/10**	-	-	-	-	-	-	-	-	-	-	-	-	-	-	-	-	-	-	-	**-**
**2010/11**	6	2	5	18	39	71	171	547	1,645	3,387	5,098	6,801	9,460	11,425	14,178	14,668	12,430	8,660	5,171	**93,782**
**2011/12**	6	3	2	10	32	50	166	551	1,587	3,279	5,358	6,662	9,555	11,239	13,471	14,559	12,963	8,765	5,507	**93,765**
**2012/13**	14	3	-	10	32	48	181	463	1,515	3,316	5,226	6,614	8,880	11,374	13,251	13,825	12,442	8,444	5,402	**91,040**
**2013/14**	12	3	5	9	40	52	169	519	1,391	3,158	5,254	6,767	8,661	11,648	12,561	13,702	11,915	7,987	5,261	**89,114**
**Stroke**																				
**2000/01**	57	25	33	86	107	236	406	501	685	1,186	1,658	1,989	2,720	3,957	5,850	8,924	9,165	8,681	5,132	**51,398**
**2001/02**	61	21	38	86	129	203	331	560	794	1,142	1,696	2,007	2,493	3,780	5,792	8,512	9,693	8,455	5,372	**51,165**
**2002/03**	76	35	39	67	112	229	347	633	888	1,225	1,707	2,210	2,596	3,807	5,811	8,399	10,200	8,697	5,901	**52,979**
**2003/04**	75	30	57	60	131	198	341	603	783	1,227	1,575	2,284	2,557	3,780	5,463	8,094	10,435	8,059	6,005	**51,757**
**2004/05**	77	46	115	60	119	190	363	586	944	1,332	1,612	2,225	2,700	3,803	5,246	7,904	10,652	7,730	6,081	**51,785**
**2005/06**	80	35	39	95	127	207	331	618	938	1,195	1,648	2,251	2,644	3,712	5,522	7,745	9,849	8,240	6,370	**51,646**
**2006/07**	83	47	59	86	137	196	331	633	885	1,322	1,640	2,149	2,563	3,376	5,010	7,211	9,493	8,282	6,095	**49,598**
**2007/08**	114	46	45	66	124	235	313	569	998	1,344	1,573	2,134	2,852	3,346	4,920	6,976	8,930	8,489	6,065	**49,139**
**2008/09**	215	99	104	163	277	458	620	1,171	2,079	2,868	3,386	4,205	5,895	7,166	10,382	14,444	18,114	19,076	12,154	**102,876**
**2009/10**	-	-	-	-	-	-	-	-	-	-	-	-	-	-	-	-	-	-	-	**-**

**2010/ 11**	107	54	71	92	158	230	310	571	992	1,525	1,918	2,207	3,090	3,845	5,387	7,311	9,401	9,532	6,903	**53,704**
**2011/ 12**	92	62	64	94	157	199	347	519	1,098	1,640	1,880	2,297	3,053	3,835	5,543	7,440	9,269	9,385	7,317	**54,291**
**2012/ 13**	112	75	66	83	142	249	326	516	973	1,536	2,002	2,274	3,088	4,011	5,308	7,442	9,413	9,093	7,636	**54,345**
**2013/ 14**	141	72	72	81	159	211	349	564	962	1,572	2,056	2,250	2,987	4,255	5,339	7,340	9,196	8,691	7,593	**53,890**

Notes: Data for 2009/10 are not available. Hospital Episode Statistics (HES) include activity ending in the year in question and run from April to March, e.g. 2012-13 includes activity ending between 1st April 2012 and 31st March 2013. A finished admission episode (FAE) is the first period of admitted patient care under one consultant within one healthcare provider. Admissions do not represent the number of patients, as a person may have more than one admission within the period. FAEs are counted against the year or month in which the admission episode finishes. Excluding day cases.

Source: HSCIC (2015). Hospital Episode Statistics (HES). Health and Social Care Information Centre. Leeds.

Prescriptions used in the prevention and treatment of cardiovascular disease, England 1981 to 2014

	Thousands (000s)													
**Prescriptions**	**1981**	**1986**	**1991**	**1996**	**2001**	**2006**	**2007**	**2008**	**2009**	**2010**	**2011**	**2012**	**2013**	**2014**
Digoxin and other positive inotropic drugs (2.1)	4,243	3,722	3,822	3,871	4,031	4,126	4,141	4,149	4,119	4,088	4,006	3,900	3,770	3,634
Diuretics (2.2)	20,678	21,996	22,195	23,106	30,203	37,582	37,355	37,536	37,511	37,687	37,563	37,258	36,650	36,208
Anti-arrhythmic drugs (2.3)	232	334	532	840	1,292	1,265	1,247	1,226	1,188	1,174	1,156	1,129	1,107	1,088
Beta-adrenoreceptor blocking drugs (2.4)	9,827	12,525	14,282	14,375	20,439	27,378	26,810	27,634	28,529	29,686	30,924	32,355	33,597	34,859
Antihypertensive and heart failure drugs (2.5)	4,912	4,424	6,431	12,125	25,047	47,742	53,634	57,823	60,838	63,571	65,449	67,184	68,652	70,071
Nitrates, calcium blockers & other antianginal drugs (2.6)	5,156	10,314	16,718	21,971	26,814	34,707	37,214	39,100	40,575	42,043	43,086	44,675	45,868	46,992
Anticoagulants and protamine (2.8)	629	900	1,356	2,609	4,609	6,790	7,309	7,991	8,546	9,157	9,773	10,723	11,906	13,173
Antiplatelet drugs (2.9)	281	1,058	3,619	9,002	18,891	32,779	35,382	38,124	39,107	38,182	38,351	38,603	38,661	38,443

Anti-fibrinolytic drugs and haemostatics (2.11)					282	327	352	358	363	373	392	396	393	408
Lipid-lowering drugs (2.12)	295	247	1,066	3,138	13,523	42,098	47,412	52,190	56,452	59,550	61,649	64,399	66,795	68,436
**All prescriptions for disease of the circulatory system**	**46,252**	**55,520**	**70,022**	**91,037**	**145,131**	**234,793**	**250,855**	**266,130**	**277,244**	**285,530**	**292,370**	**300,647**	**307,424**	**313,342**

Notes: The data up to 1990 are not consistent with data from 1991 onwards. Figures up to 1990 are based on fees and on a sample of 1 in 200 prescriptions dispensed by community pharmacists and appliance contractors only. Figures from 1991 are based on items and cover all prescriptions dispensed by community pharmacists, appliance contractors, dispensing doctors and prescriptions submitted by prescribing doctors for items personally administered. British National Formulary (BNF) codes in parentheses.

Source: Office for National Statistics (2015). Prescription cost analysis 2014. Health and Social Care Information Centre, and previous editions.

Prescriptions used in the prevention and treatment of cardiovascular disease, Wales 2005 to 2014

					**Thousands (000s)**					
**Prescriptions**	**2005**	**2006**	**2007**	**2008**	**2009**	**2010**	**2011**	**2012**	**2013**	**2014**
Digoxin and other positive inotropic drugs (2.1)	362	357	337	319	307	296	284	273	259	249
Diuretics (2.2)	3,083	3,091	3,028	3,000	2,979	2,971	2,960	2,940	2,897	2,860
Anti-arrhythmic drugs (2.3)	99	94	87	81	75	68	64	62	60	60
Beta-adrenoreceptor blocking drugs (2.4)	2,093	2,110	2,071	2,116	2,165	2,237	2,322	2,424	2,505	2,584
Antihypertensive and heart failure drugs (2.5)	3,442	3,774	4,124	4,402	4,601	4,781	4,920	5,063	5,173	5,290
Nitrates, calcium blockers & other antianginal drugs (2.6)	2,731	2,892	3,032	3,135	3,205	3,263	3,314	3,390	3,437	3,488
Anticoagulants and protamine (2.8)	576	612	650	689	723	764	808	874	944	1,030
Antiplatelet drugs (2.9)	2,563	2,716	2,846	3,011	3,045	2,933	2,905	2,890	2,859	2,816
Anti-fibrinolytic drugs and haemostatics (2.11)	23	24	28	30	30	31	33	34	33	33
Lipid-lowering drugs (2.12)	3,103	3,626	3,985	4,297	4,562	4,693	4,788	4,956	5,076	5,149
**All prescriptions for disease of the circulatory system**	**18,073**	**19,296**	**20,188**	**21,082**	**21,691**	**22,037**	**22,399**	**22,906**	**23,247**	**23,570**

Notes: British National Formulary (BNF) codes in parentheses.

Source: Health Statistics and Analysis Unit (2015). Prescription cost analysis 2014. Welsh Government: Cardiff and previous editions.

Prescriptions used in the prevention and treatment of cardiovascular disease, Scotland 2001/02 to 2014/15

					Thousands (000s)						
**Prescriptions**	**2001/02**	**2005/06**	**2006/07**	**2007/08**	**2008/09**	**2009/10**	**2010/11**	**2011/12**	**2012/13**	**2013/14**	**2014/15**
Digoxin and other positive inotropic drugs (2.1)	358	323	315	305	296	291	283	276	269	260	252
Diuretics (2.2)	3,469	3,914	3,810	3,680	3,597	3,544	3,457	3,382	3,269	3,154	3,057
Anti-arrhythmic drugs (2.3)	102	90	85	81	77	73	70	71	69	68	67
Beta-adrenoreceptor blocking drugs (2.4)	2,508	3,027	2,940	2,853	2,850	2,883	2,909	2,957	2,998	3,048	3,102
Antihypertensive and heart failure drugs (2.5)	2,298	3,777	4,127	4,462	4,693	4,875	4,965	5,045	5,095	5,160	5,220
Nitrates, calcium blockers & other antianginal drugs (2.6)	3,278	3,542	3,625	3,666	3,683	3,714	3,697	3,699	3,716	3,735	3,788
Anticoagulants and protamine (2.8)	489	612	646	676	707	743	773	819	884	958	1,035
Antiplatelet drugs (2.9)	2,461	3,448	3,545	3,652	3,743	3,724	3,577	3,506	3,404	3,295	3,218
Anti-fibrinolytic drugs and haemostatics (2.11)	35	36	38	39	41	42	43	44	47	47	47
Lipid-lowering drugs (2.12)	1,667	3,649	4,081	4,376	4,624	4,826	4,875	4,861	4,907	4,977	5,024
**All prescriptions for disease of the circulatory system**	**16,667**	**22,418**	**23,212**	**23,791**	**24,312**	**24,716**	**24,649**	**24,660**	**24,657**	**24,703**	**24,764**

Notes: British National Formulary (BNF) codes in parentheses.

Source: ISD Scotland (2015). Prescription Cost Analysis 2014/15. NHS National Services: Edinburgh and previous editions.

Prescriptions used in the prevention and treatment of cardiovascular disease, Northern Ireland 2000 to 2014

					Thousands (000s)						
**Prescriptions**	**2000**	**2005**	**2006**	**2007**	**2008**	**2009**	**2010**	**2011**	**2012**	**2013**	**2014**
Digoxin and other positive inotropic drugs (2.1)	132	107	104	101	99	96	95	93	92	90	87
Diuretics (2.2)	819	1,015	1,009	992	983	983	992	1,002	996	978	960
Anti-arrhythmic drugs (2.3)	41	38	36	35	34	32	31	32	31	30	29
Beta-adrenoreceptor blocking drugs (2.4)	641	908	918	915	937	967	1,020	1,059	1,108	1,150	1,189
Antihypertensive and heart failure drugs (2.5)	602	1,145	1,247	1,364	1,445	1,517	1,595	1,640	1,689	1,728	1,770
Nitrates, calcium blockers & other antianginal drugs (2.6)	928	1,001	1,039	1,080	1,089	1,097	1,124	1,154	1,188	1,218	1,233
Anticoagulants and protamine (2.8)	138	183	194	207	220	233	249	274	300	334	365
Antiplatelet drugs (2.9)	539	957	1,026	1,095	1,151	1,177	1,192	1,223	1,239	1,239	1,228
Anti-fibrinolytic drugs and haemostatics (2.11)	0	12	12	13	13	14	14	16	17	16	16
Lipid-lowering drugs (2.12)	376	1,047	1,227	1,393	1,534	1,652	1,761	1,838	1,901	1,954	1,988
**All prescriptions for disease of the circulatory system**	**4,226**	**6,413**	**6,812**	**7,195**	**7,505**	**7,769**	**8,073**	**8,331**	**8,560**	**8,736**	**8,869**

Notes: British National Formulary (BNF) codes in parentheses.

Source: HSC (2015). Prescription Cost Analysis 2014. Business Services Organisation: Belfast.
